# Insight into the genetic variability analysis and cultivar identification of tall fescue by using SSR markers

**DOI:** 10.1186/s41065-016-0013-1

**Published:** 2016-08-11

**Authors:** Kaixin Fu, Zhihui Guo, Xinquan Zhang, Yan Fan, Wendan Wu, Daxu Li, Yan Peng, Linkai Huang, Ming Sun, Shiqie Bai, Xiao Ma

**Affiliations:** 1 Animal Science and Technology College, Sichuan Agricultural University, Chengdu, 611130 China; 2 Chongqing Municipal Institute of Animal Husbandry, Chongqing, 400039 China; 3 Sichuan Academy of Grassland Science, Chengdu, 611731 China

**Keywords:** Tall fescue, SSR markers, Genetic diversity, Cultivar identification, Principal component analysis, Neighbor joining, Population structure

## Abstract

**Background:**

Genetic diversity of 19 forage-type and 2 turf-type cultivars of tall fescue (*Festuca arundinacea* Schreb.) was revealed using SSR markers in an attempt to explore the genetic relationships among them, and examine potential use of SSR markers to identify cultivars by bulked samples.

**Results:**

A total of 227 clear band was scored with 14 SSR primers and out of which 201 (88.6 %) were found polymorphic. The percentage of polymorphic bands (PPB) per primer pair varied from 62.5 to 100 % with an average of 86.9 %. The polymorphism information content (PIC) value ranged from 0.116 to 0.347 with an average of 0.257 and the highest PIC value (0.347) was noticed for primer NFA040 followed by NFA113 (0.346) whereas the highest discriminating power (D) of 1 was shown in NFA037 and LMgSSR02-01C. A Neighbor-joining dendrogram and the principal component analysis identified six major clusters and grouped the cultivars in agreement with their breeding histories. STRUCTURE analysis divided these cultivars into 3 sub-clades which correspond to distance based groupings.

**Conclusion:**

These findings indicates that SSR markers by bulking strategy are a useful tool to measure genetic diversity among tall fescue cultivars and could be used to supplement morphological data for plant variety protection.

## Background

*Festuca arundinacea* Schreb., commonly known as tall fescue is the predominant cool-season perennial grass that is widely grown throughout the temperate regions of the world [1]. It forms the forage basis for beef cow and calf production and also is widely used as turf in lawns, parks, football fields, highway medians, and roadsides [2]. It is a cross-pollinated allohexaploid (2n =6× = 42) with the genomic constitution PPG_1_G_1_G_2_G_2_ and a high degree of self-incompatibility [3, 4]. These features make breeding efforts generally focus on the development of superior synthetic cultivars or improved heterogeneous populations, both of which consist of genetically unique individuals that share many characters [5, 6]. Simultaneously, the relative drought tolerance of tall fescue and climatic changes are leading to increased cultivation in the transitional climates between temperate and subtropical zones [7]. The demand for forage type cultivars of tall fescue has resulted in the development and release of hundreds of cultivars since 1940s [7].

The accurate description of genetic diversity in natural and artificial populations and identification of elite tall fescue cultivars rely on molecular techniques since morphological traits are easily vulnerable to environmental conditions and often showed the limited efficiency of detection of inter-varietal and intra-varietal polymorphisms on account of environmental plasticity [8]. Molecular marker analysis offers an efficient approach for assessing genetic diversity and germplasm characterization in cross-pollinated grass species [9, 10]. In view of cheapness and effectiveness, bulking strategy has been successfully employed to investigate genetic variation between cultivars of allogamous species [11]. Additionally, a value of 20 genotypes per population seems appropriate for most foragegrass species [12]. RFLP and AFLP have been used to distinguish several cultivars of tall fescue by pooled DNA samples [8, 13], but these methods require more technically demanding skills than conventional PCR markers [14]. Simple sequence repeats (SSR) have become one of the most widely used molecular marker systems in plant genetics due to the co-dominant inheritance, relative abundance, multi-allelic nature, extensive genome coverage, high reproducibility, and simple detection [15]. A large number of SSR markers have been developed for the *Lolium*/*Festuca* complex species, such as tall fescue [15, 16] and *L. multiflorum* [17]. Moreover, it has been reported that SSR markers has showed a high transferability across closely related species [18].

To date, the old cultivar Kentucky-31 and its derived cultivars bred in North America have been widely introduced to other parts of the world as a forage base for beef, wool and dairy production [19]. However, there is little information about the genetic relationships between these popular cultivars. The objective of the present work, therefore, was (i) to reveal patterns of molecular polymorphism and (ii) to survey the extent of genetic variability and relationships among some elite cultivars of tall fescue from North America and Europe by using SSR markers and bulked samples. Here we wanted to confirm known genetic relationships among these cultivars based on recorded pedigrees and to evaluate the usefulness of SSR markers for germplasm identification.

## Methods

### Plant material and DNA extraction

Nineteen forage- and two turf-type cultivars of tall fescue (*F. arundinacea* Schreb.) were included in this experiment, all of whose seeds were provided by National Plant Germplasm System of USDA. Pedigree or breeding history information of 21 cultivars could be traced, listed in the Table 1. Two of all cultivars, viz., Willamette and Carefree belong to turf-type, while the rest are forage-type. Seed was germinated at 26 °C on blotter paper in a lighted growth chamber. Bulked samples were composed by DNA extraction from pooled tissue samples consisting of single leaves from each of the 20 randomly selected plants, for each cultivar. Total genomic DNA was isolated from bulked leaves according to the protocol of using a modified CTAB method [20]. The quality and concentration of the extracted DNA were determined by Nano-Drop ND 2000 spectrophotometer (NanoDrop Technologies, Inc.) and 1 % (w/v) agarose gels electrophoresis. The isolated genomic DNA was diluted to 10 ng/μL and stored at −20 °C for use.

**Table 1:** Description of the plant material analyzed

Cultivar	Country	Origin/Pedigree	Registration date
Kenhy	U.S.	11 42-chromosome *Lolium multiflorum* × *F. arundinacea* clones	1977
Cajun	U.S.	cultivar AuTriumph	1989
Maximize	U.S.	ecotypes from southeast France	1993
Kentucky 31	U.S.	ecotype from temperate pasture in Kentucky	1972
Kenwell	U.S.	three inbred lines	1968
Alta	U.S.	a 4-year-old plant selection in Oregon.	1945
Fawn	U.S.	temperate 8-clone synthetic	1974
Martin	U.S.	2 clones from broad based population.	1987
Missouri-96	U.S.	13 clones from France germplasm	1979
Forager	U.S.	Kenwell, Fawn, Kentucky 31, etc.	1980
Barcel	Netherland	13 temperate clones from Netherlands	1981
Johnstone	U.S.	blend of two strains of Kenhy derivatives and 42-chromosome *Lolium* sp. × *F. arundinacea* hybrid clones	1983
Au triumph	U.S.	an open pollinated population AF-5 comprised of 12 genotypess	1983
Willamette	U.S.	an open pollinated progeny of 5 elite parental clones	1985
Safe	U.S.	Kenhy and two germplasm with diseases and drought resistance	1985
Barvetia	Netherland	temperate 13-clone synthetic	unavailable
Mozark	U.S.	Kenmont, Kentucky-31, and introductions from Algeria and France	1987
Penngrazer	U.S.	Kentucky 31	1988
Cattleclub	U.S.	Kentucky 31	1988
Carefree	U.S.	Houndog, Rutgers, and GPTF accessions.	1989
Nanryo	Japan	Fawn, Kentucky 31, Rozelle, Electa, Krasnadorskaja and Yamanami	2006

### SSR maker primers and SSR-PCR amplification

A total of 15 SSR markers (Table 2) were used to characterize 21 cultivars tall fescue, of which primer LMgSSR02-01C is genic-SSR markers from annual ryegrass [17], the remaining 14 were EST-SSR markers from tall fescue [15]. Each 15 μL amplification reaction consisted of 3.0 μL of template DNA (10 ng/μL), 0.6 μL primer (5 pM), 0.3 μL of Taq polymerase (2.5 U/μL), 3 μL of sterile distilled water and 7.5 μL of 2× Taq PCR Master Mix (Tiangen Biotech, Beijing, China). Touchdown PCR amplification was carried out under the following conditions: 5 min at 95 °C; 16 cycles of 95 °C for 50 s, 68 °C with 0.5 °C decrease per cycle for 50 s until the annealing temperature reached 60 °C, and 72 °C for 1 min; 19 cycles of 95 °C for 50 s, 60 °C for 50 s, 72 °C for 1 min; followed by a final extension at 72 °C for 10 min, and in the end 4 °C as a holding temperature. The PCR amplification products were electrophoresed in 8 % non-denaturing polyacrylamide gel at 150 V for 30 min, followed by 2 h at 350 V. Then the gels were visualized by silver staining f and photographed by a gel scanner.

**Table 2:** Characteristics of 15 SSR markers selected for use in the study

Primes	motif	Size range (bp)	NTB	NPB	PPB (%)	PIC	I	CSM	DV	D
LMgSSR02-01C	(aac)_26_	86–193	24	23	95.8	0.252	0.403	3	21	1.000
NFA018	(cgg)_6_	136–198	10	9	90	0.267	0.411	2	12	0.952
NFA021	(cct)_7_	191–358	14	12	85.7	0.259	0.402	1	12	0.971
NFA022	(agg)_6_	180–391	15	13	86.7	0.269	0.410	3	13	0.967
NFA035	(ggc)_6_	177–371	16	10	62.5	0.193	0.293	3	8	0.914
NFA037	(acc)_6_	177–384	25	24	96	0.279	0.434	4	21	1.000
NFA040	(cgg)_6_	233–404	12	12	100	0.347	0.518	1	15	0.986
NFA050	(cag)_7_	187–361	8	5	62.5	0.116	0.205	1	1	0.267
NFA060	(tag)_8_	205–350	13	12	92.3	0.237	0.383	2	13	0.967
NFA065	(agc)_6_	200–344	18	18	100	0.298	0.461	3	19	0.995
NFA066	(cct)_6_	165–267	11	9	81.8	0.250	0.380	2	11	0.962
NFA113	(cgg)_6_	161–357	19	18	94.7	0.346	0.514	1	19	0.995
NFA133	(cgg)_7_	162–268	16	15	93.8	0.274	0.424	3	15	0.967
NFA150	(ctg)_7_	173–297	13	10	76.9	0.306	0.443	1	12	0.971
NFA153	(gca)_7_	187–294	13	11	84.6	0.170	0.286	5	9	0.900
Total	—		227	201	88.6	—	—	35	21	1
Mean	—		15.1	13.4	86.9	0.257	0.398	2.3	13.4	0.920

*NTB* number of total bands, *NPB* number of polymorphic bands, *PPB* percentage of polymorphic bands, *PIC* polymorphic information content, *I* Shannon’s information index, *CSM* Cultivar-specific markers (presence and absence), *DV* Distinguished varieties, *D* discriminating power

### Data analysis

In spite of co-dominant nature of SSR, there is great difficulty in allele calling by amplified pattern because of the allohexaploid nature of tall fescue and the bulking strategy used in present study. Therefore, unequivocally scorable bands were scored manually as either present (1) and absent (0) to create the binary raw data matrix for further analysis. The total number of bands (TB) and polymorphic bands (PB), polymorphic rate (P), Shannon information index (I) [21], and polymorphism information content (PIC) [22] were calculated as MS Excel 2010, using the formula: PIC = 1-∑P^2^_ij_, where P_ij_ is the frequency of the *j*th allele (marker) for the *i*th SSR locus. The distinguished varieties (DV), which were calculated by the number of unique sequences required to identify a particular cultivar from the clustering dendrogram of all 21 cultivars. To compare the efficiency of the markers in varietal identification, the discrimination power (D) [23] was estimated for each primer. This parameter was calculated in accordance with the formula as follows: $D=1-{\displaystyle {\sum}_{i=1}^I pi}\frac{\left(Npi-1\right)}{N-1}$, where D is the probability that two randomly selected samples have different and distinct banding patterns, *p*_*i*_ is the frequency of the *i*th pattern revealed by each primer, N is the number of samples analyzed and *I* is the total number of patterns generated by each primer [23]. The presence/absence data matrix was further used to calculate genetic similarity (GS) between pairs of cultivars according to Dice’s similarity coefficient using NTSYSpc v2.2 [24]. Then a genetic distance (GD) matrix developed as GD = 1-GS was used to construct dendrograms based on the neighbour-joining (NJ) clustering method with a bootstrapping value of 10,000 replications by the FreeTree software [25]. To verify the adjustment between genetic distance matrices and respective dendrogram-derived matrices (cophenetic matrix), the cophenetic correlation coefficient (r) was estimated by NTSYSpc v2.2. As well as the principal coordinate analysis (PCoA) was obtained by the similarity matrix, which was computed from the same program. Further, Bayesian model-based cluster analysis was performed to infer genetic structure and to define the number of clusters in the data set using the software STRUCTURE version 2.3.4 [26]. The membership of each cultivars was tested for the range of genetic clusters from K = 1 to 10 with admixture model and without prior information on their origin. Twenty independent runs were assessed for each fixed K and each run consisted of 20,000 burn-in period and 50,000 MCMC iterations. The most likely value of K was determined by examination of the ΔK statistic and L(K) [27] using Structure Harvester [28]. A consensus STRUCTURE plot was obtained from the admixture repeats using the greedy algorithm in CLUMPP version 1.1 [29], and final plots were produced in STRUCTURE PLOT [30]. Within a subgroup, cultivars with inferred ancestry based on probability values ≥60 % were assigned to a different group, and those with <60 % were treated as “admixture”, i.e., these cultivars seem to have a mixed ancestry from parents belonging to different origins or gene pools.

## Results and discussion

### Statistical analysis of SSR markers

Due to the outbreeding nature of tall fescue, we used bulked samples that represent the mixture of genotypes with a cultivar. In the present work, genetic variability among the 21 tall fescue cultivars was analyzed using a set of SSR primer pairs (PPs), most of which originated from ESTs sequence of tall fescue [15]. Out of 100 original primer pairs (PPs), 15 selected PPs could generate reproducible and polymorphic patterns among the cultivars, and hence these 15 PPs were retained for further statistical analyses. Totally 227 bands were produced, of which 201 (88.6 %) were polymorphic with an average of 13.4 polymorphic bands per primer (Table 2). The number of scorable bands produced per PPs ranged from 8 (NFA050) to 25 (NFA037) with an average of 15.1, and amplicon size varied from 80 to 400 bp. The percentage of polymorphism varied between 62.5 (NFA035 and NFA50) to 100 (NFA40 and NFA65) with an average value of 86.9 %.

The polymorphism information content (PIC) was in the range from 0.116 to 0.347 with an average value of 0.258 and PIC value was found to be highest with the primer NFA40 (0.347), followed by NFA113 (0.346) and NFA150 (0.306). Nine SSR primers showed more PIC than the average value (0.257). The Shannon index ranged from 0.205 to 0.518 and NFA40 showed highest diversity (0.518), followed by NFA113 (0.514); and average Shannon index was 0.398. Furthermore, the number of the evaluated cultivars distinguished by any SSR marker ranged from 1 to 21, with an average of 13.4 per PPs. The discriminating power (D), a measure of the efficiency of a primer or a locus is an effective method to know the ability of a primer in distinguishing the crop genotypes [31]. In this study, D value ranged between 0.267 (NFA50) to 1 (NFA37 and LMgSSR02-01C) with an average value of 0.921 (Table 2). Here, 14 out of 15 SSR markers showed high discriminatory power (>0.90), indicating that these 15 PPs used in this study were highly effective in tall fescue cultivar characterization. Primer NFA37 and LMgSSR02-01C, which showed the highest D value being one, was found to be the best SSR markers for detecting polymorphism in tall fescue. Although only 35 cultivar-specific markers were observed for 15 primers, all of 21 cultivars had their own unique SSR banding patterns individually, which could be used to distinguish cultivars by characteristic sets of bands.

### Dice’s distance coefficient and hierarchical clustering

Two hundred and twenty-seven fragments from 15 SSR loci were used to estimate pairwise Dice’s distances among 21 tall fescue cultivars. The Dice’s coefficient was moderately high, varying from 0.163 (Forager vs. Fawn) to 0.475 (AuTriumph vs. Barvetia), with an average of 0.320 (Table 3). These low values were similar to those obtained from studies about analyzing tall fescue cultivars and/or populations by RFLP [8] and AFLP [13], which were less than 0.50 among cultivars. The less genetic distance indicated a relatively low genetic diversity or a closer relationship among studied cultivars although some visible differences obviously exist between them. This is not surprising considering the breeding histories of these cultivars (Table 1). Some popular cultivars such as Kentucky 31, often served as common parental germplasm to intercross with other limited germplasm in breeding program of many tall fescue cultivars [8]. Therefore, the similar pedigree or germplasm sources could account for the high genetic similarity and close relationship among cultivars evaluated. Likewise, seven perennial ryegrass cultivars representing a broad germplasm sample also showed high levels of genetic similarity using SSR markers [32]. Besides that, this low level of genetic diversity could be due in part, to the tall fescue’s allogamy and self-incompatibility [3], as well as to its hexaploid composition [33]. Typically, the cross-pollinated species maintain relatively high intra-population variability as compared to its inter-population variability [12, 34]. The Analysis of molecular variance (AMOVA) indicated that most (92.3 %) of the molecular variation in wild Iranian tall fescue populations exists among individuals within populations, with lesser amounts among populations (7.7 %) [35]. As a result, the enormous intra-cultivar genetic variability led to the high genetic similarity among tall fescue cultivars.

**Table 3:** Dice’s distance matrix of 21 cultivars of tall fescue based on SSR profile

	1	2	3	4	5	6	7	8	9	10	11	12	13	14	15	16	17	18	19	20	21
Kenhy	0																				
Cajun	0.336	0																			
Maximize	0.313	0.295	0																		
Kentucky31	0.291	0.302	0.352	0																	
Kenwell	0.289	0.292	0.304	0.237	0																
Alta	0.294	0.245	0.243	0.306	0.268	0															
Fawn	0.345	0.291	0.326	0.354	0.311	0.253	0														
Martin	0.415	0.418	0.322	0.384	0.364	0.322	0.307	0													
Missouri96	0.313	0.295	0.327	0.192	0.294	0.289	0.348	0.379	0												
Forager	0.366	0.293	0.316	0.343	0.313	0.257	0.163	0.285	0.306	0											
Barcel	0.372	0.382	0.330	0.347	0.359	0.298	0.317	0.364	0.286	0.329	0										
Johnstone	0.219	0.370	0.340	0.288	0.256	0.272	0.341	0.361	0.310	0.340	0.322	0									
AuTriumph	0.429	0.254	0.292	0.355	0.356	0.293	0.297	0.348	0.379	0.218	0.301	0.403	0								
Willamette	0.280	0.312	0.296	0.275	0.303	0.268	0.326	0.393	0.265	0.326	0.273	0.288	0.370	0							
Safe	0.262	0.330	0.385	0.194	0.217	0.327	0.348	0.367	0.268	0.337	0.373	0.230	0.388	0.317	0						
Barvetia	0.345	0.431	0.449	0.387	0.367	0.380	0.377	0.361	0.440	0.398	0.406	0.365	0.475	0.408	0.360	0					
Mozark	0.337	0.248	0.340	0.265	0.271	0.294	0.319	0.326	0.321	0.271	0.354	0.333	0.306	0.359	0.312	0.405	0				
Penngrazer	0.320	0.257	0.324	0.265	0.272	0.305	0.290	0.341	0.294	0.282	0.380	0.327	0.317	0.333	0.285	0.390	0.243	0			
Cattleclub	0.337	0.350	0.330	0.188	0.245	0.260	0.360	0.358	0.248	0.330	0.303	0.277	0.360	0.292	0.240	0.445	0.281	0.273	0		
Carefree	0.356	0.316	0.339	0.265	0.327	0.313	0.320	0.422	0.274	0.340	0.395	0.371	0.370	0.321	0.376	0.447	0.307	0.300	0.333	0	
Nanryo	0.345	0.330	0.294	0.344	0.344	0.295	0.190	0.256	0.326	0.200	0.317	0.352	0.277	0.326	0.358	0.389	0.289	0.301	0.330	0.289	0

**Fig. 1:**
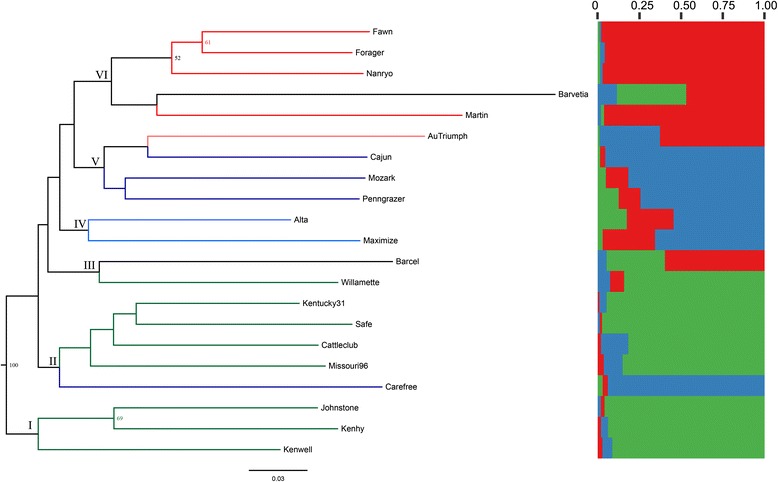
NJ dendrogram of 21 tall fescue cultivars based on Dice’s coefficient and Model-based Bayesian clustering performed in STRUCTURE for K = 3 populations. Each color represents one population and length of colored segment shows estimated membership proportion of each cultivars. (For interpretation of the references to color in this figure legend, the reader is referred to the web version of this article)

A dendrogram was generated from the NJ cluster analysis of SSR data (Fig. 1). This dendrogram divided the 21 culitvars into six major clades in general agreement with their breeding origins with a few exceptions. A high correlation (*r* = 0.79, *P* < 0.01) between the cophenetic matrix and the original matrix revealed a high goodness of fit for the cultivars clustering. Clade 1 consisted of three cultivars, Kenhy, Johnstone and Kenwell. Here, both Kenhy and Johnstone origined from derivatives of 11 42-chromosome *Lolium* × *Festuca* hybrid clones at Kentucky Agricultural Experiment Station (KAES) of USA [36–38]. Kenwell was also developed from three inbred lines at KAES and may provide share some common pollen sources with Kenhy and Johnstone [39], therefore it is not surprising that it was also included in Clade 1. Clade 2 was composed of Kentucky 31, Safe, Cattleclub, Missouri 96 and Carefree, whose clustering placement could be chiefly attributed to their pedigree origin. Kentucky 31 originated from a population that had undergone approximately 43 years of natural selection on a farm in Kentucky State of USA [37, 40]. And Cattleclub originated from several old Kentucky 31 seed fields by phenotypic selection [41]. However, Missouri-96 was derived from plant introductions from France [42]. It was used as a pollen source in the development of Houndog, one of the first improved turf-type cultivar [37]. Moreover, six parental clones from Houndog was used to ploycross with other germplasm in the development of Carefree, a turf-type synthetic [41], which is the most dissimilar from the others found in Clade 2. Yet it is hard to explain the low distance of 0.194 between Safe and Kentucky 31, since Safe was derived from Kenhy existing in Clade 1 [41]. Clade 3 was randomly made up of Barcel and Willamette supported by a bootstrap value of 47 %, indicating that these two cultivars are distinctly different from the other cultivars in the study. Barcel derived from old pastures in the Netherlands [41], but there is little information available concerning the parentage of Willamette other than it may have some common germplasm with cultivar Houndog [37]. Clade 4 contained two cultivars Alta and Maximize with a high pairwise similarity of 0.76, while it is hard to give a clear elucidation by their pedigree history. Alta was developed from a plant selection in Oregon [43], and Maximize was resulted from French ecotypes [44]. Clade 5 included four cultivars, viz., AuTriumph, Cajun, Mozark and Penngrazer. By and large, this clade came together as predicted by the breeding histories for these cultivars. AuTriumph was developed from 12 introduction genotypes and Cajun derived from AuTriumph [45, 46], while Penngrazer could trace to Kentucky 31, one of the four parent clones of Mozark was also Kentucky 31 [47]. However, Penngrazer and Mozark failed to group tighter with the Kentucky 31 in the Clade 2. The reason for that probably is that the nature of outcrossing and multiple mass selections resulted in an unequal contribution of parent plants in the final genetic makeup of a cultivar [48]. Clade 6 including five cultivars could be divided into sub-clades with three and one cultivar respectively. Here Forager and Nanryo sharing one of the common parent germplasm Fawn resulted to a firm genetic relationship with the high bootstrap values [49, 50]. Additionally, Barvetia and Martin formed a sub-clade with a middle bootstrap value of 42 %, but this could not correspond to their pedigree information, since Barvetia was developed in Netherland and Martin was developed in Missouri university of USA [8, 47, 51]. Yet it was worth noting that most of NJ clusters showed lower bootstrap values below the 50 %, which suggested that more primers may be needed to identify genetic relationship between cultivars surveyed. In particular, the detailed pedigree information of some commercial or patented cultivars is desperately needed to explain the genetic relationship among different cultivars. In short, these results illustrated that SSR markers were effective in surveying the affinities among tall fescue cultivars studied.

**Fig. 2:**
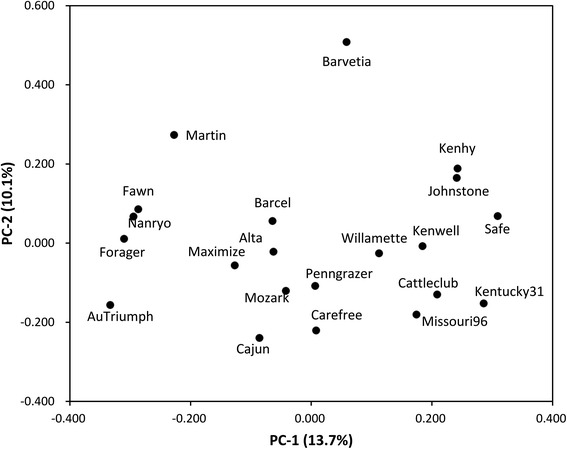
Principal coordinates analysis (PCoA) scatterplot of 21 tall fescue cultivars based on the first two principal coordinates

The principal coordinate analysis (PCoA) was done to see the displacement of the cultivars and to further confirm the clustering pattern obtained from the dendrogram (Fig. 2). The first three eigenvectors accounted for 32.3 % of total variation among all the cultivars. The first and second axes represented 13.7 and 10.1 % of the variation respectively. The affinities produced by PCoA are generally in agreement with the NJ cluster and Dice’s dissimilarity coefficients analyses. Some cultivars shared common parental resources were closely dispersed in the PCoA plot, as Nanryo, Forager and Fawn showed closely related each other on NJ dendrogram.

### Population structure

The pattern of population structure was further analyzed with a Model based Bayesian approach implemented in Structure program. We investigated the range from K = 1 to K = 10 and calculated the posterior probability for each value of K using the estimated log likelihood of K. The number of clusters best fitting the data was K = 3 as indicated by the modal value of ΔK. As a result, the studied cultivars were successfully assigned to three subgroups with slight mixing (K = 3, ΔK = 65.37) as represented in Fig. 1 and Table 3; inferred clusters were calculated with more than 60 % probability intervals. In general, the clustering based on Bayesian statistics was found to be in consistency with distance based NJ clustering, whereas there were deviations and fragmentation of genetic clusters because of admixed cultivars such as Alta, Barvetia and Barcel. A second analysis of each cultivar assignment probabilities for the model K = 3 shows that cluster 1 (red) is corresponding to the Clade 6 and Barcel in Clade 3 in NJ dendrogram. The cluster 2 (blue) includes Clade 4 and 5, and Carefree in Clade 2 of NJ tree. Finally, cluster 3 (green) consists of Clade 1, remaining entries except Carefree and Willamette in Clade 3. Moreover, the affinities produced by PCoA are generally in agreement with the results of the STRUCTURE analysis, since the PCoA grouped the cultivars into three clusters strongly differentiated, which correspond substantially to three inferred sub-clusters from STRUCTURE analysis (Fig. 1). It’s worth noting that cultivars Alta, Barvetia and Barcel showed the admixed membership based on Q-matrix values, nor did they reflected clear membership in any of the groups identified in the PCoA. This might be due to the fact that extensively used poly-intercross among genotypes from registered varieties or exchanged germplasm in breeding programs led to disturbed Hardy-Weinberg equilibrium (HWE) and genetic admixture in the synthetic cultivar populations [7].

In this study, due to using a DNA bulking strategy, it was possible to survey the genetic variation among the 21 tall fescue cultivars regardless of intra-cultivar variability. Assaying bulked samples not only drastically reduces the number of individual samples that need to be processed [52], but also results in a preferential elimination of rare alleles by dilution in larger bulk samples [10, 53] and therefore simplifies the marker profile of an individual cultivar or accession. In previous studies, various molecular markers have been used to determine diversity among heterogeneous cultivars populations of outcrossing fodder species based on bulked DNA samples [7, 11, 12, 54–56]. Likewise, the 21 tall fescue cultivars used in this study were easily characterized using SSR markers. The large number of molecular markers produced in this study showed that SSR patterns based on bulked DNA samples were found to be a fast, reliable, and highly efficient method to analyze genetic relationships among heterogeneous cultivars of tall fescue. These results will help breeders choose cultivars more genetically distant to be used in crosses in order to obtain transgressive segregation of some agronomic trait in the hybrid descendant populations.

## Conclusions

In conclusion, SSR patterns based on bulked DNA samples were found to be a good method of choice to evaluate the extent of genetic variability and relationships among 21 heterogeneous tall fescue cultivars. Even though low levels of diversity were detected here, most of SSR markers showed extremely high discriminatory power for evaluated cultivars. SSR analysis is therefore a powerful tool for distinguishing synthetic cultivars as well as assessing genetic relationships among cultivars in outcrossing grass species.

## Abbreviations

AFLP, amplified fragment length polymorphism; AMOVA, analysis of molecular variance; CTAB, hexadecyl trimethyl ammonium bromide; D, discriminating power; DV, distinguished varieties; GD, genetic distance; GS, genetic similarity; I, Shannon information index; NJ, neighbour-joining; P, polymorphic rate; PB, polymorphic bands; PCoA, principal coordinate analysis; PCR, polymerase chain reaction; PIC, polymorphism information content; PIC, the polymorphism information content; PPB, the percentage of polymorphic bands; PPs, primer pairs; RFLP, restriction fragment length polymorphism; SSR, simple sequence repeats; TB, total number of bands
